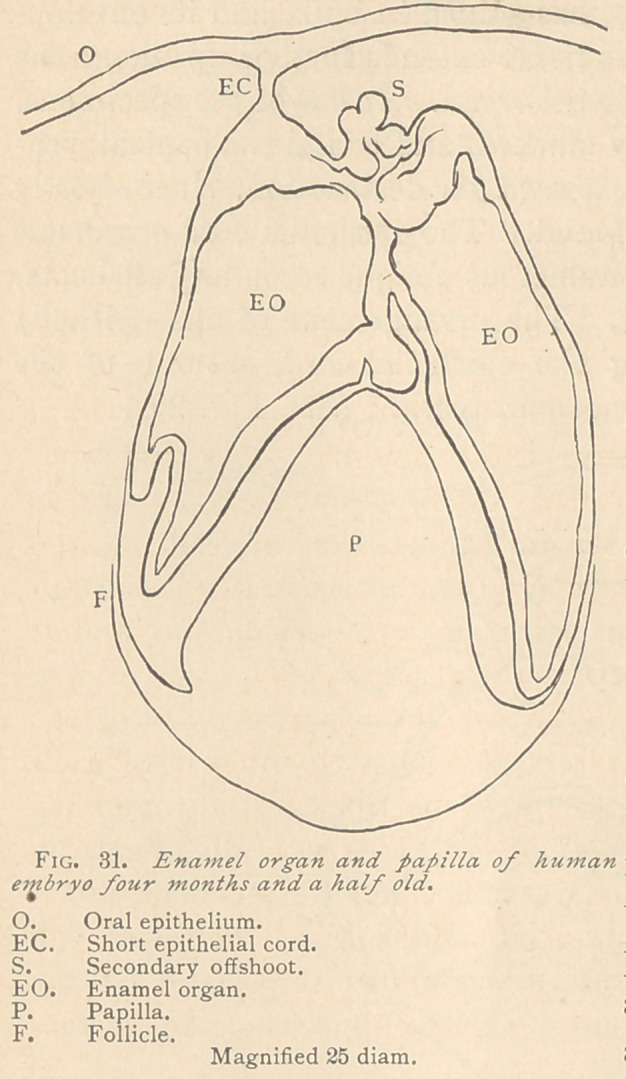# Contributions to the History of Development of the Teeth

**Published:** 1887-09

**Authors:** Carl Heitzmann, C. F. W. Bödecker


### Independent Practitioner.

VOL. VIII. SEPTEMBER, 1887. NO. 9.

NOTE.—No paper published or to be published in another journal will be accepted for this department. All papers must be in the hands of the Editor before the first day of the month preceding that in which they are expected to appear. Extra copies will be furnished to each contributor of an accepted original article, and reprints, in pamphlet form, may be had at the cost of the paper, press-work and binding, if ordered when the manuscript is forwarded. The Editor and Publishers are not responsible for the opinions expressed by contributors. The journal is issued promptly, on the first day of each month.

### Original Communications.

### HISTORY OF THE DEVELOPMENT OF THE TEETH.

BY CARL HEITZMANN, M. D., AND C. F. W. BÖDECKER, D. D. S., M. D. S.

CONTINUED FROM PAGE 402.

### IV. THE EPITHELIAL CORD OF THE ENAMEL ORGAN.

The history of the development of the tissues of the teeth is not intelligible on the basis established by the researches of Remak. According to this observer, the epiblast and the hypoblast are epithelial formations, and give rise to epithelial tissues and their derivations only. The mesoblast is connective tissue, including the muscles, the blood-vessels and the lymph-vessels. Early investigators in embryology were embarrassed in attempting to explain the origin and formation of the central nervous system (brain and spinal cord), which unquestionably has its origin in the epiblast, although in full development it contains a large amount of connective tissue, and blood-vessels, which are intermixed with nerve substance proper, such as the gray substance, the ganglionic corpuscles, and the axis cylinders. For that portion of the epiblast which gives rise to the nerve-centers, some authors have proposed the name "neuro-epithelium." This would imply that from an original epithelial structure, tissues may arise which have no resemblance to and do not contain epithelia, except in the ventricular lining of the brain and the central canal of the spinal cord. It is admitted, therefore, that a certain portion of the epithelium of the embryo contains protoplasm which, in turn, becomes gray substance, forms the ganglionic corpuscles, and axis cylinders as well as the investment of the latter—the myeline or nerve fat—and the perineurium or neuroglia, which all admit is a delicate fibrous connective tissue.

The history of the development of the enamel likewise furnishes striking proofs of the fact that the theory of exclusiveness, so far as the epiblast is concerned, is not tenable. The writers have demonstrated that the original epithelial cord of the enamel organ serves for carrying a certain amount of building material into the depth of the connective tissue. This material, however, loses its epithelial nature as soon as it gives rise to the enamel organ proper, which is myxomatous connective tissue. The fact was established that the tissue termed enamel is epithelial in its origin only, and changes its character shortly before its appearance. We must admit that enamel will not be formed unless upon an epithelial basis, the epithelium in this instance being the conveyor of the protoplasm from which enamel originates. But to assert that enamel is an epithelial structure throughout would be as erroneous as to call the brain and the spinal cord epithelial structures.

From an organo-genetic point of view, we may say that the outer senses of the animal organism, serving for perceptions from the outer world, are formations of the outer investment of the animal, its epiblast. The brain, being the highest perfection of sensual perception, retains its origin from the epiblast. The same may be said of the teeth, which, in some lower order of amphibious organisms, such as chelonia, are nothing but horny ledges, or a thickening of the epithelium. Even at the height of development they retain their genesis from the epiblast, and are, at least as far as the enamal is concerned, derivations from it.

Let us now consider the direction taken by the epithelial cord of the enamel organ into the depth of the connective tissue, up to the time when the enamel organ is ready for the formation of the enamel.

The first trace of the future tooth in the human embryo is visible about the sixth week of intra-uterine life, when the epithelium of the oral cavity is as yet little developed. Here we notice a furrow, which is situated close behind the lip, and is succeeded by an elevation of medullary tissue. (See Fig. 21.)

Fig. 21. *Human embryo six weeks old. Frontal section.*

T. Tongue.

L. Lip.

B. Base of oral cavity.

F. Furrow, which is in the transvere section, funnel shaped.

Magnified 25 diam.

After this period follows the formation of an epithelial peg, appearing, not at the bottom of the primitive dental furrow, but at some distance from the latter. This peg appears as a reduplication of the epithelial layer covering the elevation behind the furrow. (See Fig. 22.)

Fig. 22. *Base of oral cavity of human embryo two months old. Frontal section.*

T. Tongue.

L. Lip.

B. Base of oral cavity.

F. Furrow.

EH. Epithelial hill.

EP. Epithelial peg.

Magnified 25 diam.

Shortly afterward, the epithelial hill has gained in height considerably, and from the point which connects the hill with the rest of the oral epithelium, the original peg has elongated into an epithelial cord. A striking feature of this cord is that from its periphery arise blunt or slightly pointed offshoots, while at the same time its distal end is noticeably broadened, the epithelia being arranged in radiating tracts throughout, but most markedly in the club-shaped enlargement of the distal end. (See Fig. 23.)

In the third month of embryonal life the epithelial hill still remains a prominent formation. From the point of its junction arises the epithelial cord, which varies, to some extent, both in width and in its course. Sometimes the cord runs nearly parallel with the base of the oral cavity, becoming devious toward its club-shaped distal end. Its periphery is slightly fluted, and from its lower contour arise scanty but strongly marked epithelial offshoots, the significance of which is not perfectly plain. We may assume that a large secondary offshoot forms the epithelial cord of a future permanent tooth, but as to the significance of the short secondary offshoots we only can suggest that the epithelium primarily producing the cord at first assumed a direction which afterward was changed.

This much is certain, that such short secondary offshoots perish and disappear in the course of further development. It would certainly be a bold hypothesis to consider all such short secondary offshoots germs of supernumerary teeth, or of third dentitions. They are too common as compared with the rare cases in which supernumerary teeth are found. At this stage of development the first trace of the papilla (the future dentine) is noticeable. (See Fig. 24.) Sometimes the epithelial cord is broad, exhibiting comparatively few blunt secondary offshoots. Its course is more or less vertical, into the depth of the connective tissue of the jaw. The epithelium within the cord is arranged into groups separated by trabeculæ somewhat resembling those of true myxomatous connective tissue. The club-shaped end of such a cord at this period shows a slight separation of the columnar epithelium into an outer and an inner layer, whereas the center of the club-shaped enlargement is occupied by medullary corpuscles, which as yet do not exhibit the characters of a myxomatous reticulum. Unquestionably, this medullary tissue has arisen from epithelia, which originally filled the club-shaped end of the cord, and it is this medullary tissue from which, soon afterward, the myxomatous reticulum of the enamel organ proper originates. (See Fig. 25.)

FIG. 25. *Floor of oral cavity of a human embryo three months old.*

EH. Epithelial hill.

F Furrow.

O. Oral epithelium.

EC. Epithelial cord.

S. Short secondary offshoot.

EO. Medullary tissue of enamel organ.

P. Papilla, detached.

Magnified 25 diam.

We found an epithelial cord of a three months embryo presenting points of interest, since it showed evidences of the germ of a temporary molar. A short offshoot arose at the place of origin of the epithelial cord, while the latter made a few shallow convolutions and then abruptly turned downward in a direction almost at right angles to its former course.

At the place of the turn a broad epithelial layer was perceptible, showing the rather thin epithelial tracts before alluded to, and in part indistinctly bordered toward the adjacent medullary tissue. The club-shaped end of the epithelial cord was divided into two segments by an intervening deep fissure. The broadest segment again showed blunt protuberances, to which corresponded shallow hills of the subjacent papilla. The club admitted of an indistinct differentiation into an external and internal epithelium, whereas its center exhibited a few faint tracts of epithelia, and a large amount of medullary tissue, which as yet had nowhere entered into the formation of a myxomatous reticulum. (See Fig. 26.)

FIG. 26. *Base of oral cavity of a human embryo three months old.*

EH. Epithelial hill.

F. Furrow.

O. Oral epithelium.

EC. Epithelial cord of enamel organ.

S. Short secondary offshoot.

SP. Broad secondary offshoot, possibly the germ of a permanent molar.

EO. Club of the epithelial cord filled with medullary tissue (the future enamel organ.)

P. Papilla.

Magnified 25 diam.

When the embryo has reached about the fourteenth week, the epithelial cord assumes considerable interest, on account of the appearance of two distinct layers at its distal end, the internal and the external epithelium, between which the myxomatous enamel organ makes its appearance. The papilla, at this point of development, has a distinct neck, being of a mushroom shape. At its distal periphery it is bordered by a thin layer of fibrous connective tissue extending upward along the external epithelium, to a certain height, and producing what has been termed the follicle, or tooth sack. In one of our specimens the epithelial cord emanates with a broad base from the epithelial hill, having at this point several short and blunt offshoots directed downward. Its general course is almost parallel to the floor of the mouth. The enamel organ originates from its distal end in an abrupt rectangular manner, with a somewhat narrow neck. The external epithelium extends into a solid peg, with a slight sigmoidal curvature, obviously the germ of the future permanent tooth. (See Fig. 27.)

FIG. 27. *Base of oral cavity of human embryo three months and a half old.*

EH. Epithelial hill.

O. Oral epithelium.

EC. Epithelial cord.

SP. Secondary offshoot of a permanent tooth, arising from the external epithelium of the enamel organ.

EO. Enamel organ of a distinctly myxomatous character.

P. Papilla.

F. Follicle.

Magnified 25 diam.

In another specimen of the same period the epithelial cord arises from the base of the epithelial hill, with a narrow neck, in immediate connection with a solid epithelial peg, running a downward vertical course, with a slight sigmoidal curvature. The epithelial cord itself shows blunt offshoots upward as well as downward, the former being characterized by a distinct concentric arrangement of their epithelia. The general course of the epithelial cord is slightly downward. Its cup-shaped distal end is marked by three prolongations, the concavities of which correspond to two myrtle-leaf shaped papillæ. Obviously, this is the germ of a future temporary molar. No trace of a corresponding permanent tooth was visible at the distal end of the epithelial cord. The external epithelium is very broad, and visible only along the broad cup. The enamel organ is narrow, but possessing a pronounced myxomatous structure. (See Fig. 28.)

The fourth month of embryonal life differs from the previous stage only as the myxomatous enamel organ gains considerably in volume, with a simultaneously marked differentiation into its two boundary layers, the external and the internal epithelium. The papilla, at this stage, likewise, has gained in bulk, and its enveloping layer of fibrous connective tissue extends further up along the convexity of the cup of the enamel organ. In one of the specimens, the epithelial hill is extremely marked, and contains a central vacuole, possibly the first step toward its destruction, since shortly afterward no trace of it is found. The epithelial cord originates with a narrow neck, and has numerous oblique secondary offshoots, mainly at its upper periphery. The arrangement of the epithelia into tracts is marked along the epithelial cord, not only of the temporary but also of the permanent tooth. (See Fig. 29.)

FIG. 30. *Floor of oral cavity of human embryo four months old.*

O. Oral epithelium.

EC. Epithelial cord.

S. Short pediculated offshoot.

SS. Secondary offshoot, lancet-shaped.

SP. Secondary peg of permanent tooth.

EO. Enamel organ.

P. Papilla.

F. Follicle.

Magnified 25 diam.

In another specimen of the same age, the epithelial hill is absent. The cord has but a limited number of offshoots, some of which are pediculated, and some have the shape of a lancet. The peg of the permanent tooth is conspicuous by its devious course. (See Fig. 30.)

With the age of four months and a half, the development of the enamel organ has still further proceeded, its myxomatous tissue is plainly marked, and the papilla has correspondingly gained in bulk. The specimen illustrated is noteworthy for its short vertical epithelial cord, which is directly in connection with the lining epithelium of the oral cavity. The secondary offshoots are but short, and no trace of a peg for the permanent tooth is visible in this section. The cup of the enamel organ is lobulated, obviously belonging to a future molar. (See Fig. 31.)

FIG. 31. *Enamel organ and papilla of human embryo four months and a half old.*

O. Oral epithelium.

EC. Short epithelial cord.

S. Secondary offshoot.

EO. Enamel organ.

P. Papilla.

F. Follicle.

Magnified 25 diam.

The further changes of the enamel organ, beginning with the fifth month of fœtal life, have been illustrated and described in our previous article on the history and development of the enamel. (INDEPENDENT PRACTITIONER of 1887, page 225.)

(TO BE CONTINUED.)

## Figures and Tables

**Fig. 21. f1:**
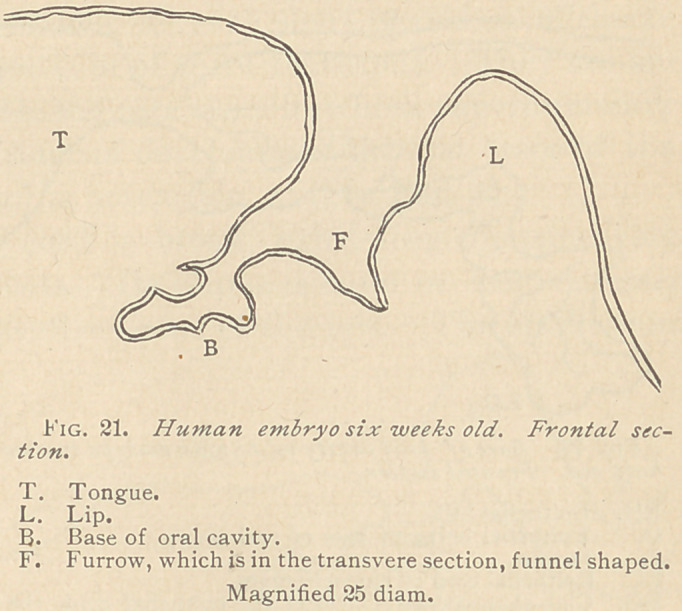


**Fig. 22. f2:**
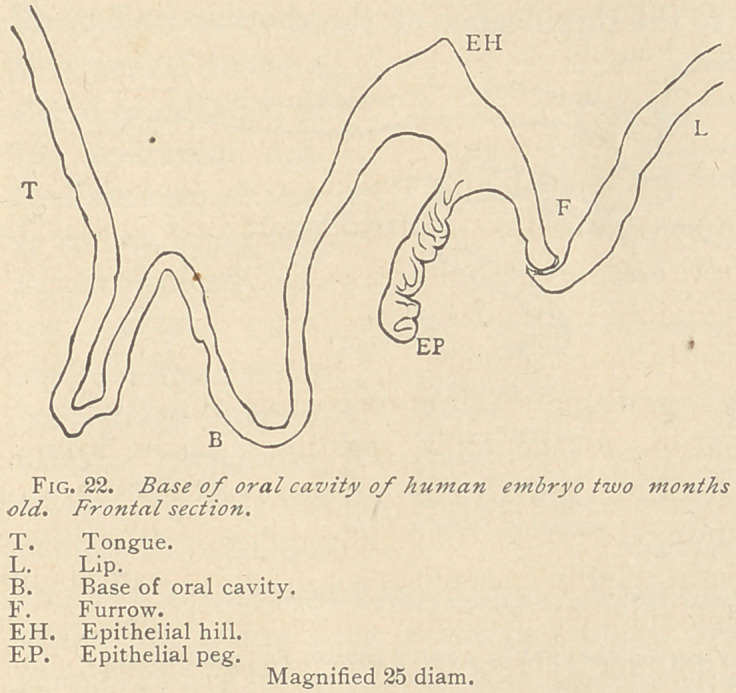


**Fig. 23. f3:**
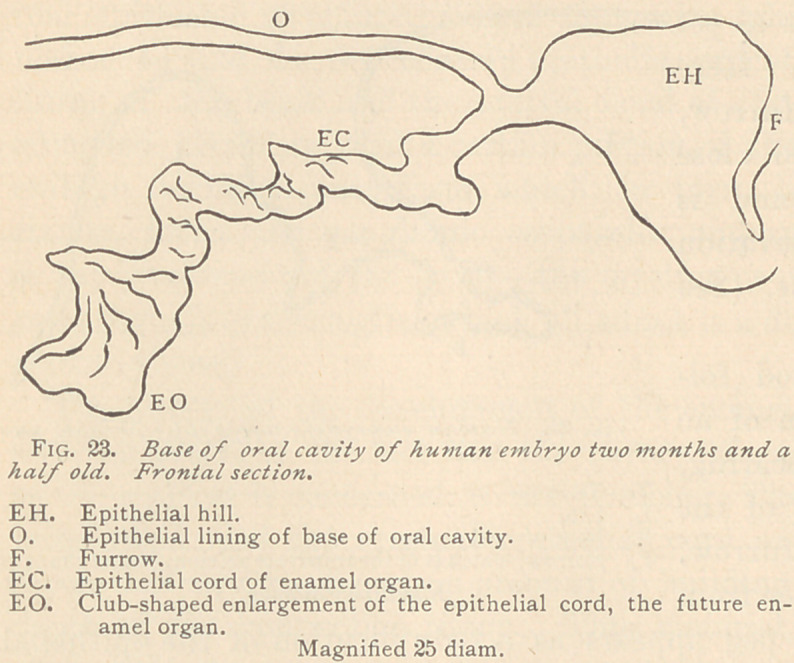


**Fig. 24. f4:**
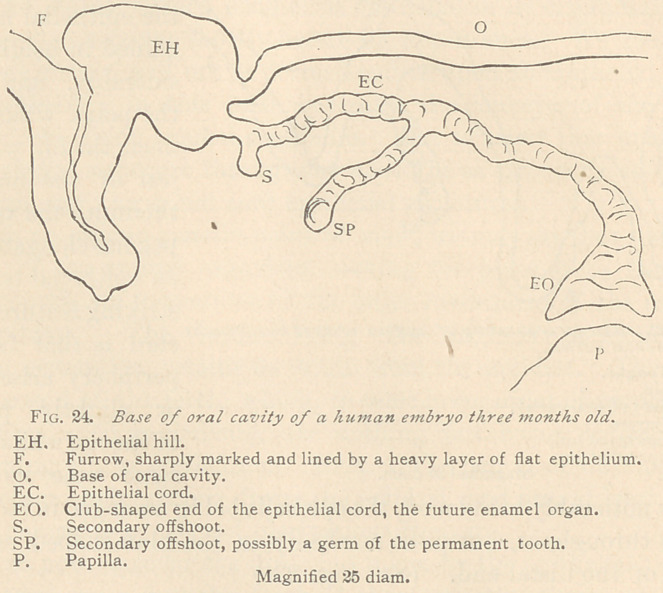


**Fig. 25. f5:**
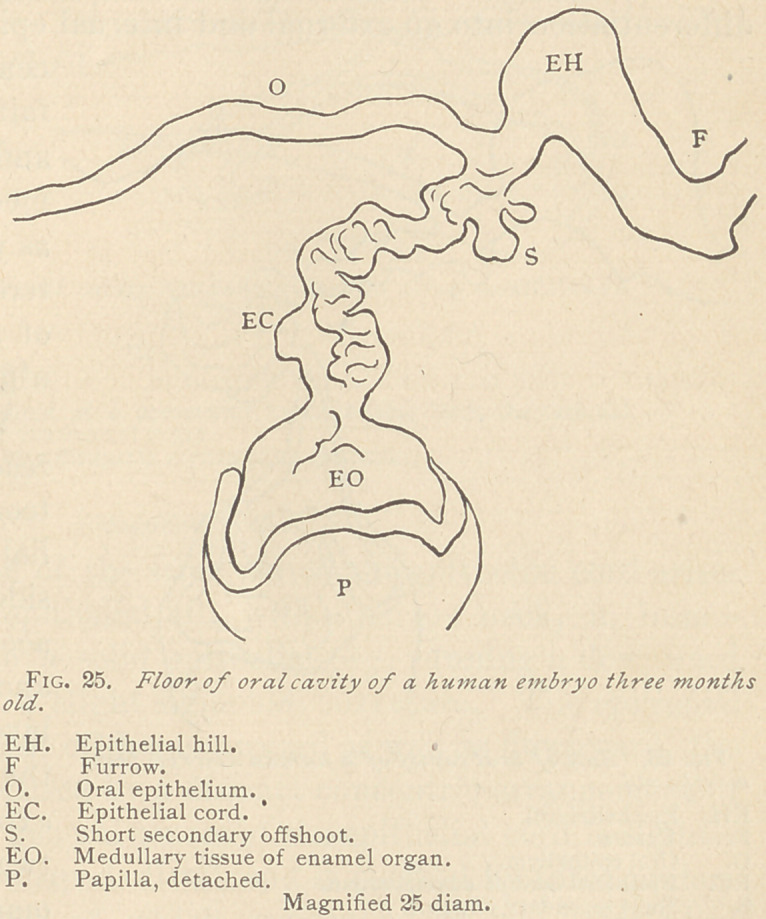


**Fig. 26. f6:**
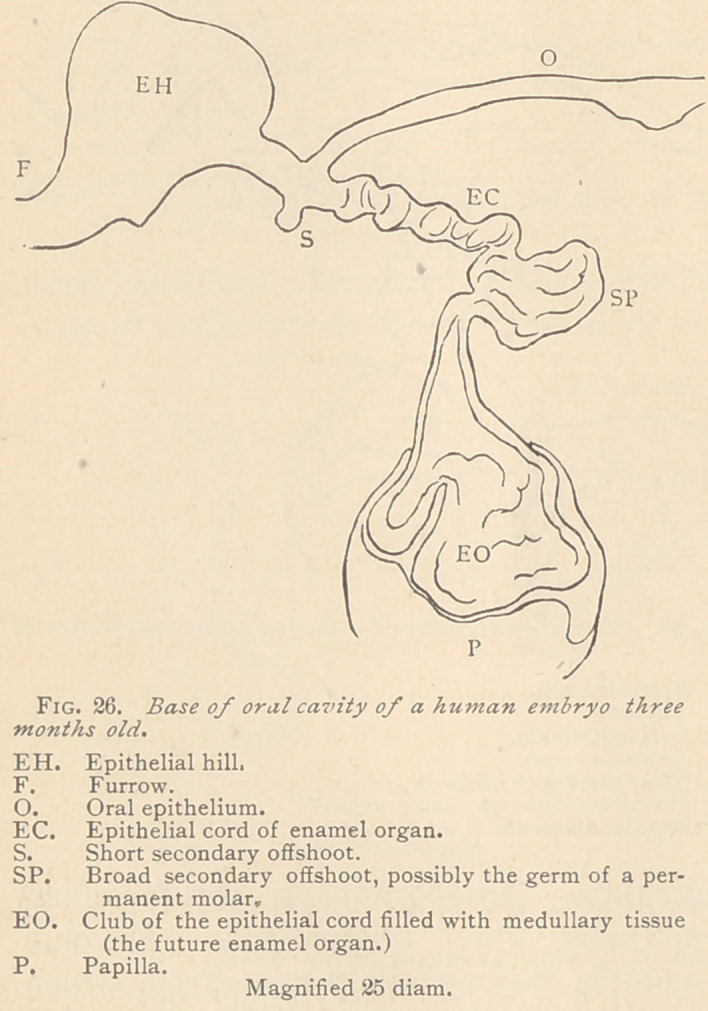


**Fig. 27. f7:**
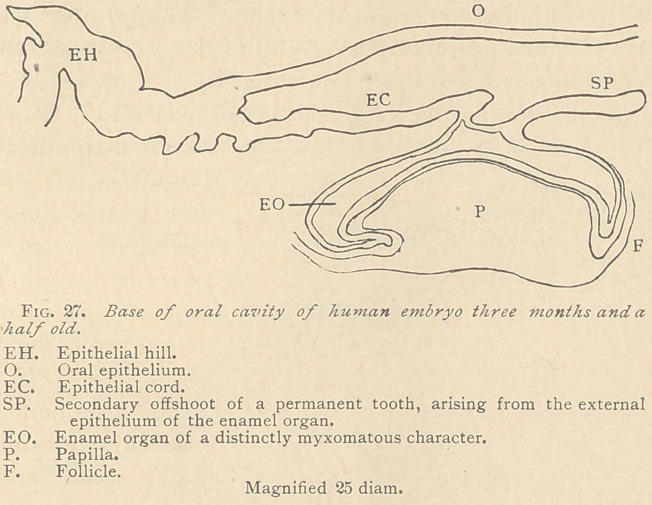


**Fig. 28. f8:**
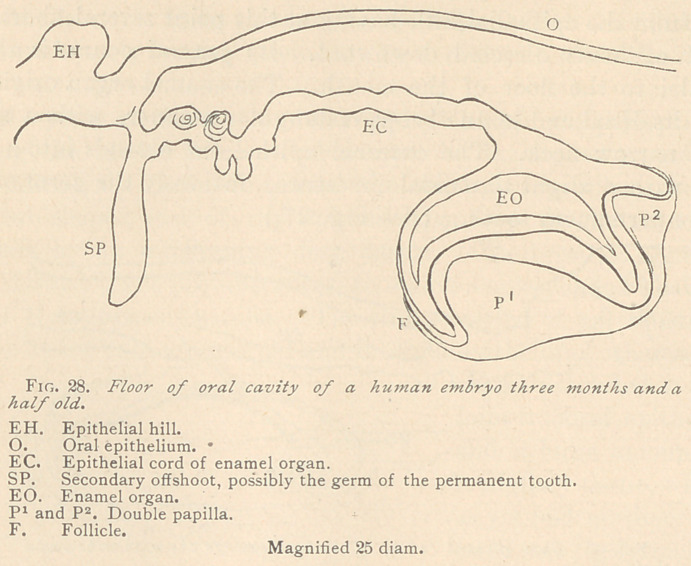


**Fig. 29. f9:**
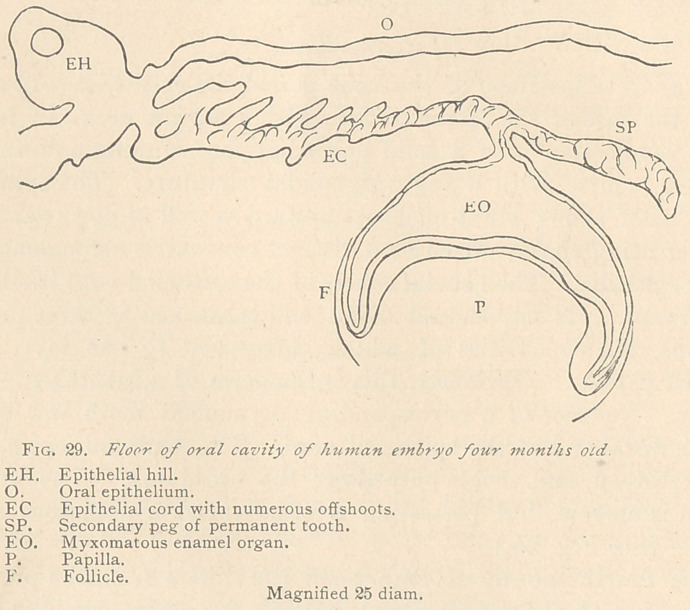


**Fig. 30. f10:**
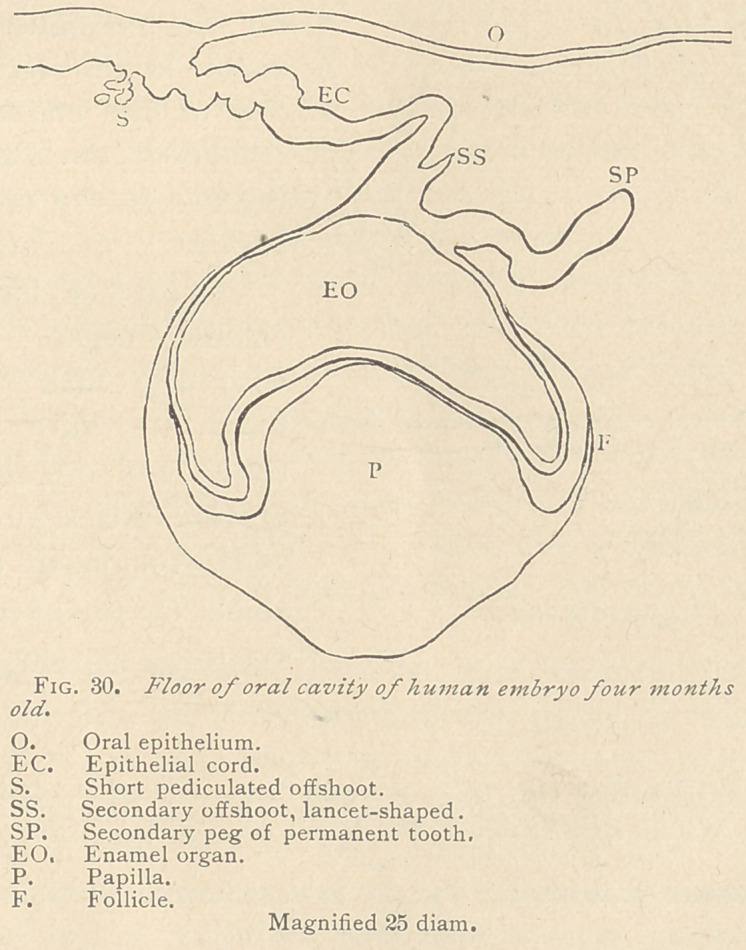


**Fig. 31. f11:**